# Address on Free Medical Schools

**Published:** 1850-01

**Authors:** 


### EDITORIAL DEPARTMENT.

---

*Address on Free Medical Schools.* By N. S. Davis, M. D., Prof. of Pysiology and Pathology in Rush Medical College.

This is a lecture introductory to the session of 1849—50, in the Rush Medical College. The author advocates the propriety of making Medical Institutions free. This, he thinks, will prove a corrective of many of the evils which now affect injuriously the character of the medical profession. He now proposes, as the great measure of medical reform, that Professors shall teach gratuitously, and that students shall be invited to enter the profession without money and without price. He states that his colleagues have so far conceded to his views, as to offer the tickets of three of the Faculty without fee. Moreover, by reference to the advertisement of the College, accompanying the address, we find that, as an additional inducement for students, approved notes will be received for the tickets of the four remaining Professors, and a deduction of $4, made to those who pay in advance.

We have no reluctance to give the Rush Medical College whatever benefit may accrue by thus aiding to circulate the above intelligence; and we leave our readers to judge, without any comments, respecting the merits of this plan of cheapening medical education, as a measure of medical reform. We opine the opinions of the author of the address will undergo some change, after a short trial of laboring without compensation. The novelty of the professorial position, and anticipations of wide-spread fame as an eloquent lecturer, may serve as incentives, for a season, but, by-and-by, the scriptural maxim that the "laborer is worthy of his hire," will be found to be not less pertinent to the medical teacher than to other laborers. The proposition on the part of the Rush medical school invites discussion in various points of view, but we forego criticism for the present.

Before reading the address of Professor Davis, we confess we had some curiosity to know how the views he had formerly expressed on the subject of education and reform, might be affected by his connection with a medical school. It is perhaps known to most of our readers that he has, of late years, been an ardent advocate of the plan of making diplomas conferred by medical collegiate institutions merely honorary distinctions, in other words, disconnecting entirely teaching, and the power of conferring a licence to practice medicine. This has been, hitherto, in his estimation, the grand desideratum in order that the standard of acquirement may be raised, and the character of the profession elevated. He appears now to have shifted his position. Medical institutions have risen so much in his favor, that young men are invited to obtain their honors and privileges, at the school with which he is connected, at a reduced price, as well as on credit. He appears to have quite forgotten the heretofore cherished project of Boards of Censors, composed of individuals unprejudiced by the duties of teaching, to decide on the qualifications of graduates, before they should be allowed to practice. It is plain, if his former views were correct, that increasing the facilities of graduation by rendering instruction cheaper, must be attended with evil results. As different methods of reform, the old and the new propositions would seem to be conflicting.

In reading the address of Dr. D., which contains some excellent remarks, our attention was arrested by the following sentence:—"Indeed, we may safely hazard the assertion, that regular, systematic clinical instruction, forms no part of the course in any medical college in America." So sweeping and broad an assertion should, certainly, not have been hazarded without information as to its correctness, and from proper inquiry the author would have ascertained that it does great injustice to *some* medical colleges to say the least. The Massachusetts General Hospital is a pretty good field for clinical instruction, and is amply available, and availed by the Massachusetts Medical College—but to go no farther than our own city, the students in attendance at the Medical Lectures, visit the hospital at stated periods, and clinical instruction is "regularly and systematically given." The same is true of St. Louis, New Orleans, Cincinnati, Louisville, although it may not perhaps be true, to the same extent, at Philadelphia, and the city of New York.

The statement contained in the sentence above quoted, is doubtless due to carelessness, but the imputation which it involves is not light, and should not have been inadvertently made.

